# A case report of acute appendicitis complicated by pylephlebitis: medical and surgical management

**DOI:** 10.1093/jscr/rjad495

**Published:** 2023-08-31

**Authors:** Krishna Patel, Jaya Sai Varre, Nate Williams, Oscar Ruiz

**Affiliations:** General Surgery, Riverside Methodist Hospital, Columbus, OH 43214, United States; General Surgery, Riverside Methodist Hospital, Columbus, OH 43214, United States; General Surgery, Riverside Methodist Hospital, Columbus, OH 43214, United States; General Surgery, Riverside Methodist Hospital, Columbus, OH 43214, United States

**Keywords:** case report, acute appendicitis, pyelephlebitis

## Abstract

Pylephlebitis is a suppurative thrombus of the portal vein and/or its branches secondary to an intra-abdominal infection. Acute appendicitis is the most common cause of emergency operation in general surgery and is typically treated with antibiotics and timely appendectomy with minimal adverse outcomes (Ferris M, Quan S, Kaplan BS, et al. The global incidence of appendicitis: a systematic review of population-based studies. *Ann Surg* 2017;**266**:237–41 and Poon S, Lee J, NG KM, Chiu GWY, et al. The current management of acute uncomplicated appendicitis: should there be a change in paradigm? A systematic review of the literatures and analysis of treatment performance. *WJES* 2017;**12**:46). Unfortunately, the identification of pyelephlebitis is difficult to make due to its nonspecific clinical presentation and can result in significant morbidity or mortality if not appropriately treated. Certain laboratory derangements and positive intra-abdominal imaging combined with a high index of suspicion can make the diagnosis. Treatment involves broad-spectrum antibiotics, anticoagulation, and source control of the primary nidus of infection. Our case presentation follows the successful clinical course of a young male diagnosed with acute appendicitis complicated by pylephlebitis. He was treated with antibiotics and anticoagulation followed by interval laparoscopic appendectomy with consequential resolution of thrombus on subsequent cross-sectional imaging.

## Introduction

The presentation of pyelophlebitis is vague, but serious consequences may result if not diagnosed and treated promptly. Early recognition on imaging is paramount, and the patient must be initiated on both antibiotic and anticoagulation treatment to permit a favorable outcome. This case report was compiled to illustrate the successful treatment of pylephlebitis from acute appendicitis with antibiotics, anticoagulation, and interval appendectomy.

## Case presentation

A previously healthy male in his 30s without any surgical history presents with 3 days of nausea/vomiting, fevers/chills, and anorexia followed by 1 day of evolving sharp epigastric to RLQ abdominal pain. He was treated a few weeks prior with antibiotics for abdominal pain in the outpatient setting. He denied any diarrhea, constipation, hematemesis, or melena. He denied any prior similar symptoms and had no prior abdominal imaging or colonoscopy.

**Figure 1:**
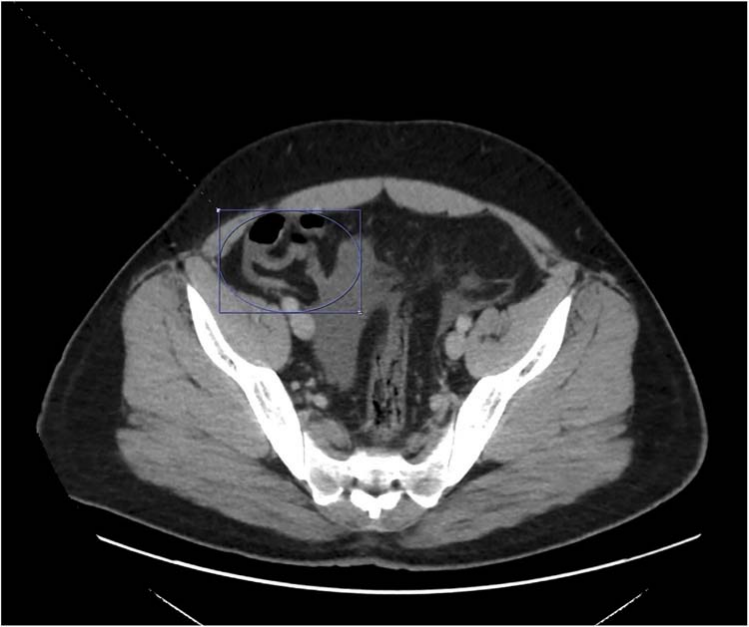
A hyperemic and inflamed appendix can be seen without an abscess, perforation, or appendicolith.

**Figure 2:**
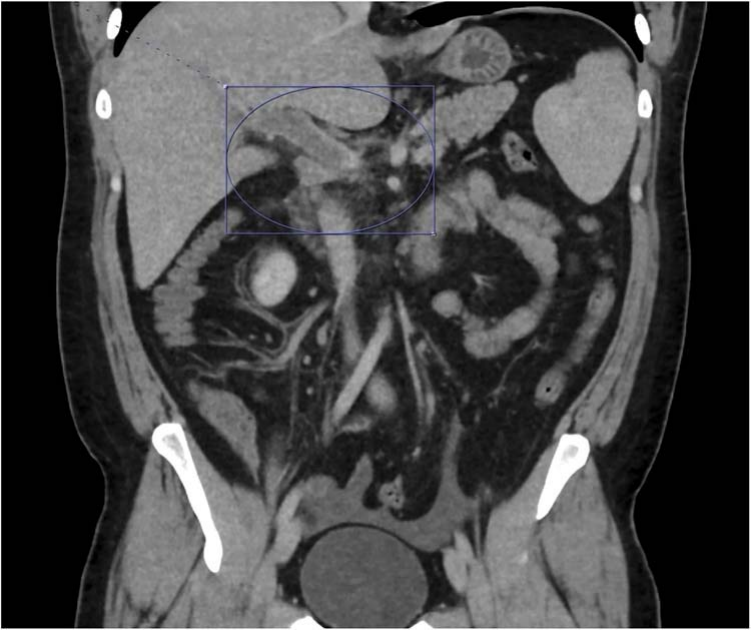
Thrombosis can be seen extending into the mesenteric venous system as well as the intrahepatic portal veins; associated mesenteritis and abdominal ascites (not well visualized) are also seen.

On presentation to the emergency department (ED), he was afebrile and normotensive with blood pressure in the 120s/80s though he had sinus tachycardia (heart rate 120–130 s). His abdomen was soft and diffusely tender with point tenderness at McBurney’s point and negative Rovsing’s sign. He had no signs of frank peritonitis. His complete blood count revealed leukocytosis (WBC 11K/μl), lactic acidosis (lactate 3 mmol/l), and mild transaminitis with AST 72 and ALT 101. An intravenous (IV) contrast-enhanced computed tomography scan of the abdomen/pelvis demonstrated a mildly dilated appendix (~8 mm) with surrounding fat stranding but no appendicolith, perforation, or abscess (Fig. 1). Interestingly, a partial portal vein (PV) and superior mesenteric vein (SMV) thrombosis were also noted (Fig. 2). Initial blood cultures were collected, which remained without growth.

Patient was admitted to the surgical service and started on broad-spectrum IV antibiotics (piperacillin–tazobactam) and was made nil per os. He was resuscitated intravenously with 2 l of crystalloid. Given the thrombosis of his PV and SMV, the patient was initiated on systemic anticoagulation with IV heparin which achieved therapeutic dosing within 24 hours. Over the course of the next few days, his abdominal pain and tachycardia improved. Interval CT abdomen/pelvis scan 5 days from presentation demonstrated progression of the thrombus to involve lobar, segmental, and subsegmental portal venous branches as well as the splenic vein distal to the portosplenic confluence (Fig. 2).

After consultation with the infectious disease team, the decision was made to transition the patient to oral antibiotics with amoxicillin/clavulanic acid for a 14-day course on discharge. The vascular medicine team was also consulted regarding the patient’s pylephlebitis. As per their recommendations, the IV heparin was transitioned to a 20-mg daily dose of rivaroxaban. The patient’s diet was then slowly advanced, and he was discharged with plans for another interval CT abdomen/pelvis scan.

**Figure 3:**
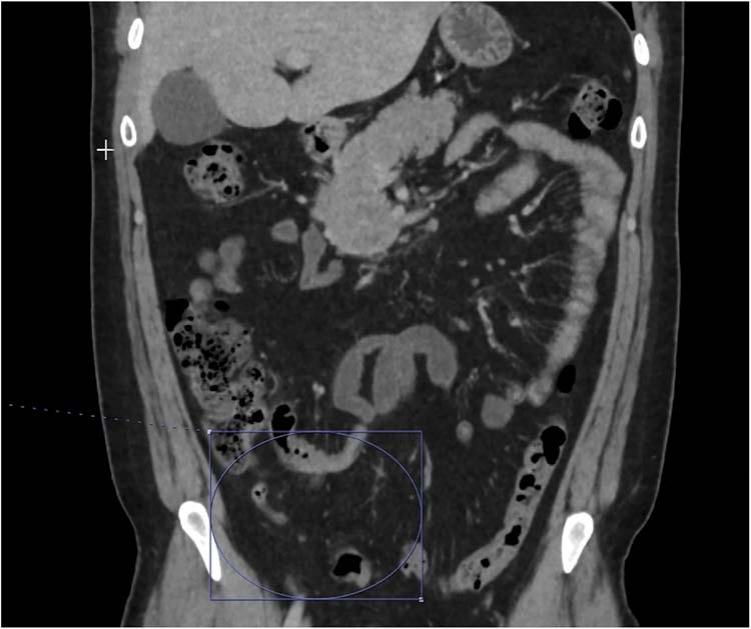
Interval decrease in inflammation surrounding the appendix.

**Figure 4:**
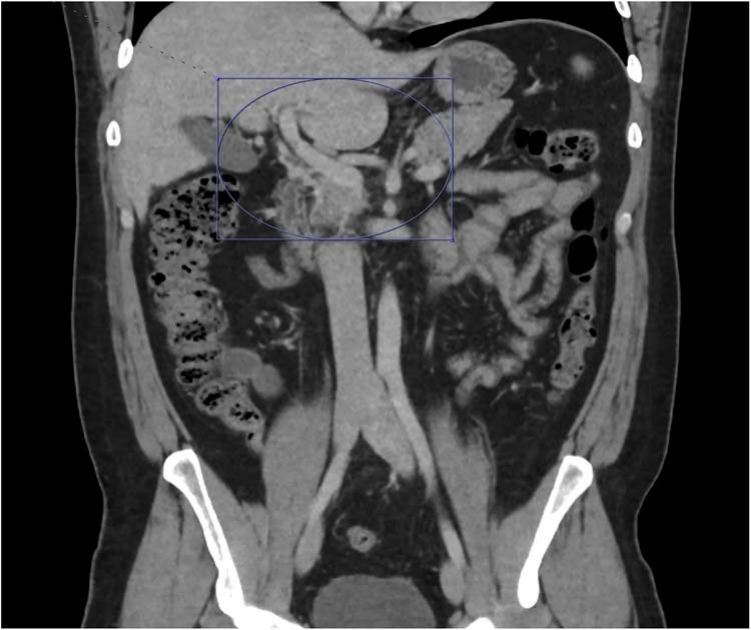
Only a slender portion of thrombus remains in the main portal vein; the previously seen splenic vein thrombosis has largely resolved (not well visualized).

At home, the patient continued to do well and remained free of abdominal pain. He was tolerating a normal diet without emesis and was having normal bowel function. His outpatient CT scan 3 months after discharge demonstrated near resolution of his subocclusive PV and SMV thrombus and attenuated periappendiceal inflammation (Figs 3 and 4). Given the patient’s clinical stability and benign radiographic findings, the patient was scheduled for a laparoscopic appendectomy.

The patient’s rivaroxaban was held 2 days prior to his surgery and his laparoscopic appendectomy was performed without complications. Patient was discharged on the same post-operative day with plans to resume his anticoagulation the subsequent day. He continued to do well postoperatively and was continued on rivaroxaban for 90 days, followed by aspirin for 1 year. His surgical pathology demonstrated no acute inflammation or malignancy within the appendix. In his last follow-up, 3 months after surgery, the patient was back to his baseline functional status. Given his uneventful post-operative course, no further abdominal imaging was pursued.

## Discussion

Pylephlebitis is a suppurative thrombus of the portal vein and/or its branches secondary to an intra-abdominal infection. Acute appendicitis is the most common cause of emergency operation in general surgery and is typically treated with antibiotics and timely appendectomy with minimal adverse outcomes. Unfortunately, nearly one fifth of all pylephlebitis cases are associated with appendicitis and results in mortality in 11–32% of cases despite maximal treatment [1, 2]. Pylephlebitis presents nonspecifically in most patients and a high index-of-suspicion must be maintained to avoid fatal complications. Given vague symptoms of presentation, early radiographic inquiry is paramount. On an intravenously contrasted CT scan, thrombus involvement of the portal system can be assessed, as well as the underlying abdominal pathology [3–5]. If pyelephebitis is suspected radiographically, the initiation of IV antibiotics and anticoagulation should be immediate [6–8]. The patient should be supported with IV fluid resuscitation as needed. A multidisciplinary approach should be undertaken with early involvement of the infectious disease and vascular medicine or hematology teams.

Pylephlebitis in the setting of intraabdominal infection develops as a result of thrombophlebitis originating in the smaller veins draining these areas of active infection. Extension into larger PVs and septic emboli into the main PV leads to pylephlebitis. PV thrombosis ultimately can lead to intestinal ischemia which portends a grave morbidity. The use of anticoagulation for treatment of pylephlebitis remains controversial as there have never been prospective randomized clinical trials that assess outcomes leading to a generalized consensus.

In our patient, IV heparin was transitioned to oral rivaroxaban (direct Xa inhibitor). Although rivaroxaban is approved for risk reduction of cerebrovascular accident in patient with nonvalvular atrial fibrillation, treatment of DVT/PE, and DVT prophylaxis in patients after hip or knee replacement, its use for treatment of pyelophlebitis remains yet to be validated by prospective randomized clinical trials [9]. As such, anticoagulation in pylephlebitis has been handled on a case-by-case basis with careful evaluation of patient-specific risk factors [10–12].

In this case, our patient was initiated on an IV heparin drip during the index hospitalization. He was discharged on rivaroxaban with interval imaging demonstrating thrombus stability. Patient subsequently had anticoagulation interrupted for interval appendectomy; however, it was resumed postoperatively for an additional 2 months with transition to ASA-81 thereafter for 1 year. Ultimately, this case report supports the use of anticoagulation in pylephlebitis similarly to previous retrospective studies which have demonstrated lower mortality with anticoagulation and increased resolution of PV thrombus [11, 12]. Nonetheless, more analysis is needed to develop a consensus regarding the preferred medication for anticoagulation as well as duration of treatment and timing for appendectomy.

## Consent

Written informed consent was obtained from the patient for publication of the case and accompanying images. A copy of the written consent is available for review by the editor of this journal.

## Conflict of interest statement

None declared.

## Funding

None declared.

## References

[1] Londono E, Hernandez D, Hernandez JD, et al. Pylephlebitis both a surgical and non-surgical pathology: a 2-case report and literature review. J Liver Res Disord Ther 2018;4:80–3.

[2] Rezac T, Zboril P, Vomáčková K, et al. A biliary tract obstruction complicated by acute appendicitis and portal vein thrombosis - a case report and review of literature. Int J Surg Case Rep 2021;84:106140. 34280969 10.1016/j.ijscr.2021.106140 PMC8274283

[3] Levin C, Koren A, Miron D, et al. Pyelephlebitis due to perforated appendicitis in a teenager. Eur J Pediatr 2009;168:633–5. 18762978 10.1007/s00431-008-0817-8

[4] Yoon S, Lee M, Jung SY, et al. Mesenteric venous thrombosis as a complication of appendicitis in an adolescent: a case report and literature review. Medicine 2019;98:e18002. 31770213 10.1097/MD.0000000000018002 PMC6890307

[5] Choudhry A, Baghdadi Y, Amr MA, et al. Pylephlebitis: a review of 95 cases. J Gastrointest Surg 2016;20:656–61. 26160320 10.1007/s11605-015-2875-3 PMC4882085

[6] Condat B, Pessione F, Helene Denninger M, et al. Recent portal or mesenteric venous thrombosis: increased recognition and frequent recanalization on anticoagulation therapy. Hepatology 2000;32:466–70. 10960436 10.1053/jhep.2000.16597

[7] Kanellopoulou T, Alexopoulou A, Theodossiades G, et al. Pylephlebitis: an overview of non-cirrhotic cases and factors related to outcome. Scand J Infect Dis 2010;42:804–11. 20735334 10.3109/00365548.2010.508464

[8] Chirinos J, Garcia J, Alcaide ML, et al. Septic thrombophlebitis: diagnosis and management. The American journal of cardiovascular. Drugs 2006;6:9–14. 10.2165/00129784-200606010-00002 16489845

[9] Xarelto (rivaroxaban) [prescribing information]. Titusville, NJ: Janssen Pharmaceuticals Inc., 2022.

[10] Hale G, Sakkal L, Galanis T. Pylephlebitis treated with apixaban. Hosp Pract 1995;47:192–5. 10.1080/21548331.2019.1670476 31545676

[11] Naymagon L, Tremblay D, Schiano T, et al. The role of anticoagulation in pylephlebitis: a retrospective examination of characteristics and outcomes. J Thromb Thrombolysis 2020;49:325–31. 31493290 10.1007/s11239-019-01949-z

[12] Duffy F, Millan M, Schoetz DJ Jr, et al. Suppurative pylephlebitis and pylethrombosis: the role of anticoagulation. Am Surg 1995;61:1041–4. 7486441

